# Black Sea outflow response to Holocene meltwater events

**DOI:** 10.1038/s41598-018-22453-z

**Published:** 2018-03-06

**Authors:** Jens O. Herrle, Jörg Bollmann, Christina Gebühr, Hartmut Schulz, Rosie M. Sheward, Annika Giesenberg

**Affiliations:** 1 Institute of Geosciences, Altenhoeferallee 1, Goethe-University Frankfurt, D-60438 Frankfurt am Main, Germany; 2 Biodiversity and Climate Research Centre (BIK-F), Senckenberganlage 25, D-60325 Frankfurt am Main, Germany; 3 Department of Earth Sciences, University of Toronto, Earth Sciences Centre, 22 Russell Street, M5S3B1 Toronto, ON Canada; 4 Institute of Geosciences, University of Tübingen, Sigwartstr. 10, 72076 Tübingen, Germany

## Abstract

During the Holocene, North American ice sheet collapse and rapid sea-level rise reconnected the Black Sea with the global ocean. Rapid meltwater releases into the North Atlantic and associated climate change arguably slowed the pace of Neolithisation across southeastern Europe, originally hypothesized as a catastrophic flooding that fueled culturally-widespread deluge myths. However, we currently lack an independent record linking the timing of meltwater events, sea-level rise and environmental change with the timing of Neolithisation in southeastern Europe. Here, we present a sea surface salinity record from the Northern Aegean Sea indicative of two meltwater events at ~8.4 and ~7.6 kiloyears that can be directly linked to rapid declines in the establishment of Neolithic sites in southeast Europe. The meltwater events point to an increased outflow of low salinity water from the Black Sea driven by rapid sea level rise >1.4 m following freshwater outbursts from Lake Agassiz and the final decay of the Laurentide ice sheet. Our results shed new light on the link between catastrophic sea-level rise and the Neolithisation of southeastern Europe, and present a historical example of how coastal populations could have been impacted by future rapid sea-level rise.

## Introduction

**Figure 1:**
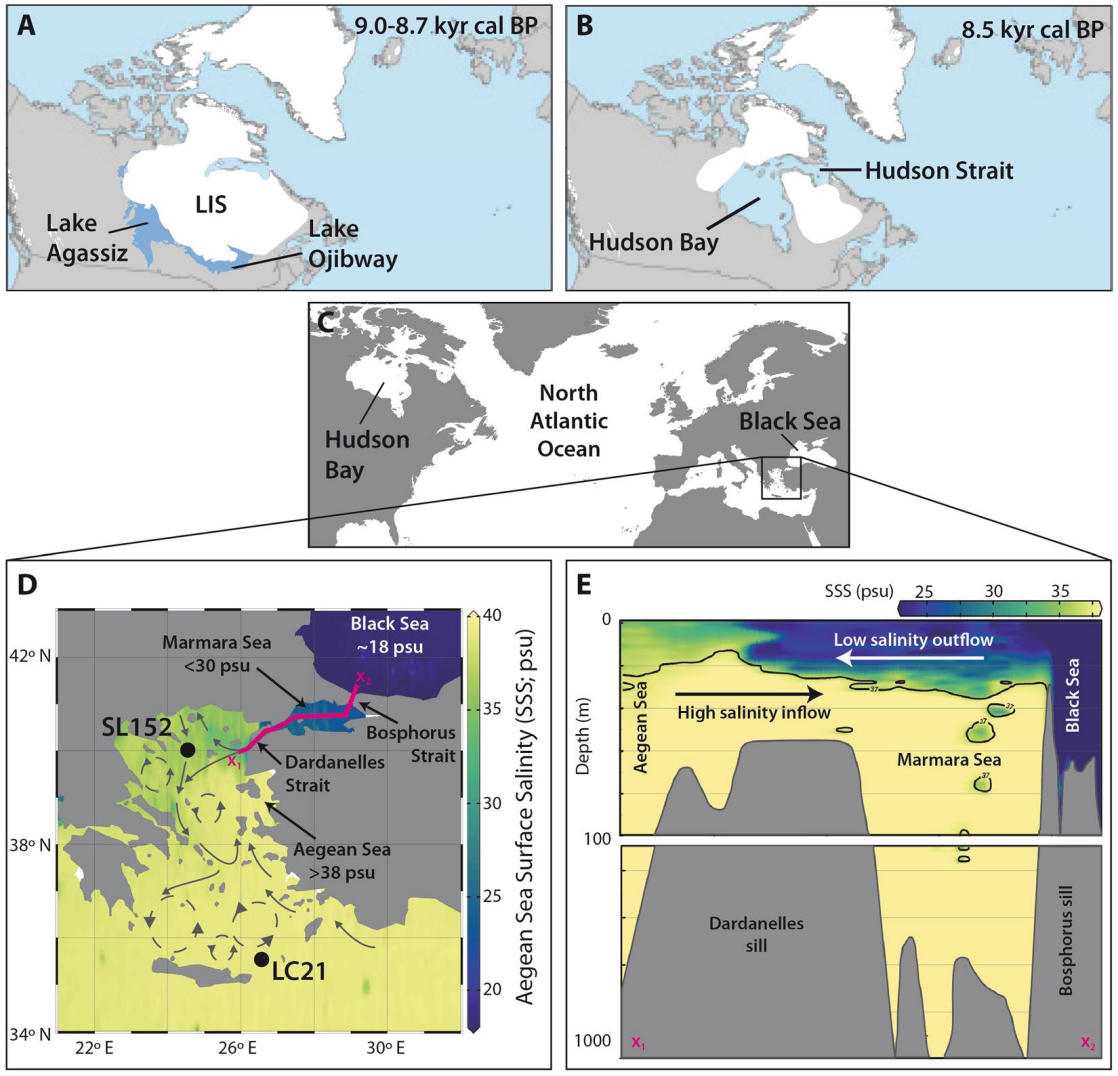
Extent of North American ice sheets during the early Holocene, location and oceanography of the studied area. (**A**,**B**) Final stage of the proglacial Lake Agassiz between about 9.0–8.7 kyrs cal BP and drainage through the Hudson Bay into the North Atlantic at about 8.5 kyrs cal BP^2^; LIS = Laurentide ice sheet modified after ref.^2^, reprinted by permission from Macmillan Publishers Ltd: Nature Geoscience (Törnqvist and Hijma, Links between early Holocene icesheet decay, sealevel rise and abrupt climate change), copyright (2012). (**C**) Northern Hemisphere map showing the studied Aegean Sea and Black Sea areas. (**D**) Location of the Aegean Sea sediment cores GeoTÜ SL152 (this study) and LC21 in relation to sea-surface salinity^35^ and the main surface water circulation patterns of the region following ref.^14^ (reprinted from Quaternary Science Reviews, volume 28, Marino et al., Early and Middle Holocene in the Aegean Sea: interplay between high and low latitude climate variability, p. 3, Copyright (2009), with permission from Elsevier. (**E**) Illustrative SSS depth profile across transect x_1_ – x_2_ (as shown on **D**) showing the present-day two-layer circulation. The maps C, D, and E are plotted using ocean data view ODV 4.7.10 (Schlitzer, R. Ocean Data View, odv.awi.de, 2017) using the salinity dataset of ref.^35^ that can be downloaded at https://odv.awi.de/data/ocean/medatlasii/.

The analysis of early Holocene episodes of rapid ice-sheet disintegration and meltwater release are highly relevant for our understanding of future sea-level change due to global warming and the associated societal effects on coastal populations^1–3^. The final drainage of the glacial Lake Agassiz in North America at about 8.4 kiloyears (kyrs) cal BP triggered a rapid sea-level rise of >1.4 m within about 200 years^2,4–7^ (Fig. 1A,B). The freshwater outburst into the North Atlantic led to a reduced thermohaline circulation causing colder and drier climatic conditions over Europe, known as the 8.2 kyrs cal BP event^4,7–10^. A similar massive meltwater event associated with a rapid sea-level rise, in the order of 4.5 m over the following <140 years in SW Sweden^11,12^, has been reported at about 7.6 kyrs cal BP but did not result in any major climate changes over Europe^2^.

**Figure 2:**
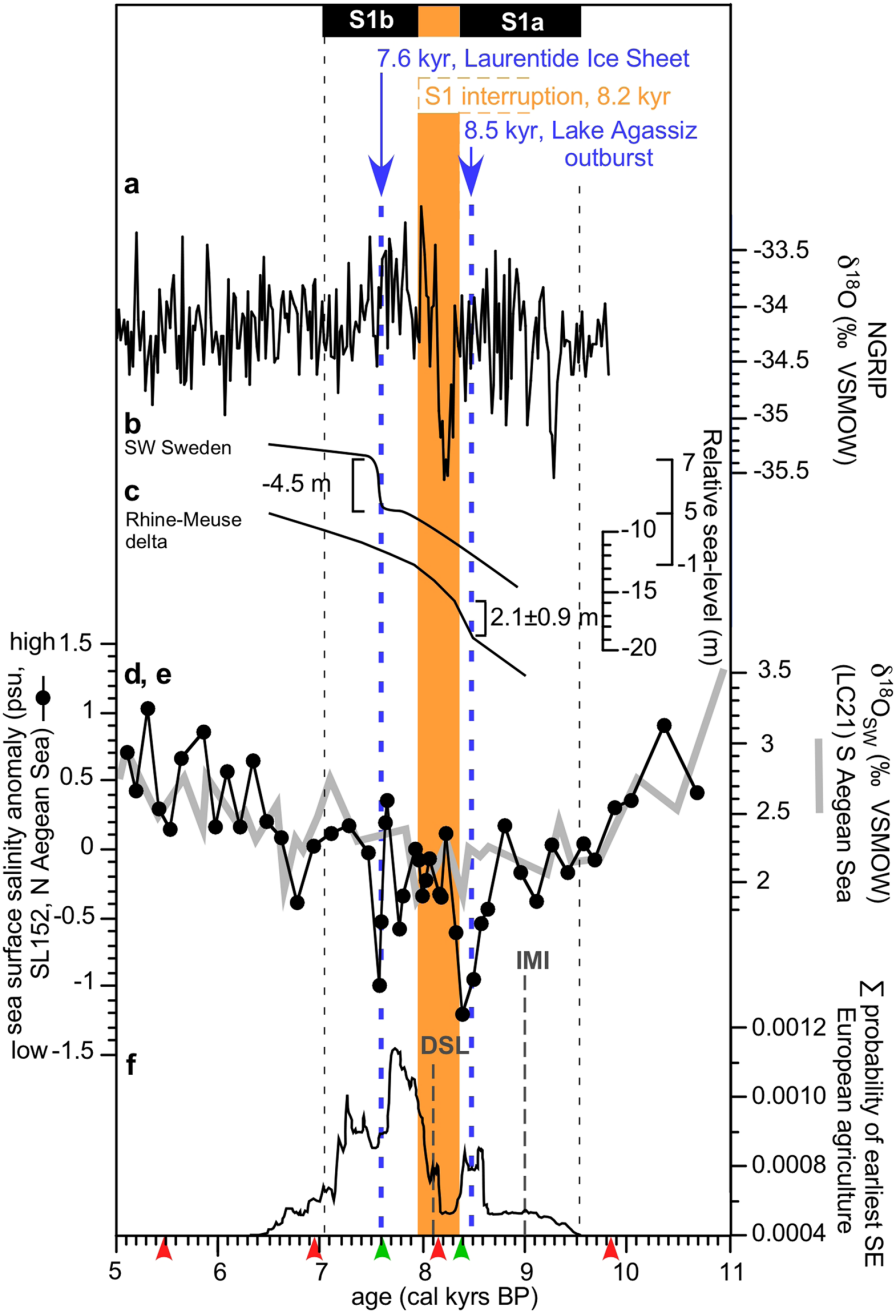
Timing of sea-level and sea surface salinity variation, and the establishment of Neolithic farmers across southeastern Europe during the Holocene. (**a**) δ^18^O record of the North Greenland ice-core^36^. (**b**) Relative sea-level changes in southwestern Sweden. (**c**) Relative sea-level change of the Rhine-Meuse-Delta^2^. (**d**) Northern Aegean Sea surface salinity (SSS) anomaly of Site GeoTÜ SL152 in practical salinity units (psu). (**e**) δ^18^O_seawater_ record from core LC21 as an indicator for fresher sea surface conditions in the southern Aegean Sea^14^. (**f**) Summed probability of earliest southeastern European agriculture^21,28^. Thin, black dashed lines indicate the duration of the sapropel 1 (S1b, S1a). The yellow bar is the interruption of the sapropel formation between about 8.0 and 8.4 kyrs cal BP in core GeoTÜ SL152. The blue dashed lines at ~7.6 and ~8.4 kyrs cal BP indicate the main phases of freshwater outburst from Lake Agassiz and the decay of the Laurentide ice sheet in North America. The grey dashed lines at 9.0 and 8.1 kyrs cal BP represent the Initial Marine Inflow (IMI) and Disappearance of the Lacustrine Species (DSL) in the Black Sea^17^. Small arrows indicate AMS dating points (green, this study and red literature data^9^ of core GeoTÜ SL152, see Method section and Supplement Table 1 and Fig. 3 for further explanations of our revised age model of GeoTÜ SL152).

We use a centennially-resolved, phytoplankton-based Sea Surface Salinity (SSS) record based on an *Emiliania huxleyi* transfer-function outlined by ref.^13^ (see Method section) from a sediment core (GeoTÜ SL152) located in the Aegean Sea approximately 130 km west from the opening of the Marmara Sea. The core is ideally located to monitor the outflow of low salinity Black Sea surface water into the Northern Aegean Sea through the Marmara Sea (Fig. 1C,D). The general trend of decreasing SSS between 11 to 5 kyrs cal BP recorded in our SSS reconstruction is remarkably mirrored in the independently-derived δ^18^O_seawater_ record^14^ from the southern Aegean Sea (core LC21, Figs 1D, 2). Both records support the view that there was an early Holocene humid phase in the eastern Mediterranean and Aegean Sea. This phase is predominantly influenced by changes in the eastern Mediterranean freshwater budget which is modulated by fluctuations in the strength of the African monsoon^14,15^. Whilst these two independent records fluctuate in concert, our new record reveals two pronounced rapid SSS drops of about 1.3 practical salinity units (psu), at ~8.4 and ~7.6 kyrs cal BP, directly dated at the minimum values to 8.4 (8314–8442 years) and 7.6 (7571–7706 years) kyrs cal BP (95% confidence intervals) (Fig. 2, see Method section, Supplement Data Table 1). These rapid salinity perturbations are strikingly synchronised to the reported timing of freshwater outbursts from Lake Agassiz and the decay of the Laurentide ice shield in North America^6,7,12^ (Fig. 2). The rapid sea level rises caused by these events led to an increased outflow of low salinity water from the Black Sea through the Marmara Sea into the Northern Aegean Sea, resulting in the two, rapid salinity drops recorded in our core. The rapid freshening of the sea surface water that is identifiable in the northernmost Aegean Sea, is absent in core LC21 in the southern Aegean Sea (Figs 1D, 2). This can be explained by the prominent influence of the Levantine Basin surface water circulation around core LC21 during the Holocene^14,16^, which would have dampened any signal of northern Aegean Sea salinity changes in the southern Aegean Sea. The increased outflow of freshwater from the Black Sea interpreted from our new salinity record is interrupted from about 8.4 and 8.0 kyrs cal BP, as evidenced by an abrupt rebound of the SSS in our record (Fig. 2). This SSS rebound corresponds to the regional expression of the cool and dry phase of the 8.2 kyrs cal BP event that was caused by a reduced thermohaline circulation well after the main Lake Agassiz’s freshwater outburst into the North Atlantic^7,9,10,14^. The dry and cold climatic conditions presumably contributed to a drop in the Black Sea lake level, leading to a reduced outflow of low salinity water from the Black Sea into the Northern Aegean Sea.

The low SSS peaks in our record are a clear indication that the present-day density-driven, two-layer circulation of low salinity surface water outflow from the Black Sea into the Marmara Sea, and vice versa, high salinity inflow of marine water into the Black Sea, was fully established no younger than 8.4 kyrs cal BP (Figs 1E, 2). Our data suggest that the two-layer circulation could have already initiated approximately 8.8 kyrs cal BP with the beginning of the steep salinity decline in our record. This indicates that the time between the Initial Marine Inflow (IMI) into the Black Sea at about 9.0 kyrs cal BP^17–19^ and the fully established two-layer circulation may have been only 200 to a maximum of 600 years. In contrast, it has been previously inferred from the disappearance of lacustrine species (DLS) in the Black Sea that the two-layer circulation was fully established at ~8.1 kyrs cal BP, 900 years after the IMI at about 9.0 kyrs cal BP^17^.

Our new data and the well constrained chronology of our core is crucial for reassessing the timing of early Holocene sea-level and climate change with the record of Neolithisation in southeastern Europe, which has been strongly debated over the last two decades^20–26^. This debate is due, in part, to the uncertainties related to the reservoir effect on ^14^C ages from the Black Sea^17,27^. The summed probability record of the earliest southeastern European agriculture, a commonly used method of demic migration of Neolithic settlements^21,28^ (Fig. 2f), shows four key features: a rapid decline of newly-established settlements at about 8.4 kyrs cal BP, followed by a low stasis until 8.2 kyrs cal BP, an increase in establishment from 8.2 to 7.7 kyrs cal BP, and a rapid decrease of settlement establishment at about 7.6 kyrs cal BP. The use of summed probability distributions of archaeological radiocarbon dates might have limitations and biases that may affect their comparison to paleoceanographic records and should therefore be used with caution^29,30^. Nevertheless, the rapid decline and the low stasis of the summed probability distribution of refs^21,28^ between 8.4 and 8.2 kyrs cal BP has previously been interpreted as an absence of Neolithic site establishments and attributed directly to a rise of the Black Sea lake-level of about 130 m due to Lake Agassiz’s freshwater outburst at about 8.5 kyr cal BP^21^, overtopping the Bosporus Sill (Fig. 1e) and leading to catastrophic flooding of the Black Sea area^21^. However, our data instead indicates that there was no catastrophic rise of the Black Sea lake level of about 130 m because the Black Sea lake level was already higher than the Bosporus sill depth as early as ~11.1–9.2 kyrs cal BP and well before Lake Agassiz’s freshwater outburst^17,23,31^, as evidenced by sediments from the Marmara Sea, the Black Sea^25,32^ and the salinity decrease in our record around 8.8 kyrs cal BP.

The timings of the steep declines in the summed probability of settlements (Fig. 2f) correspond to two rapid sea-level rises of 1.4 m and up to 4.5 m at ~8.4 and ~7.6 kyrs cal BP, respectively, recorded at SW Sweden and the Rhine-Meuse Delta^2,12^. The timing of the former sea-level rise can be attributed to freshwater outburst from Lake Agassiz and the latter to the collapse of the Laurentide ice sheet^2^ (Fig. 2). Whilst the ~8.4 kyr cal BP event has been well-documented, the second interruption in Neolithic farming establishment at ~7.6 kyrs cal BP has been not reported by previous studies. Furthermore, we relate the stasis in summed probability of agriculture between 8.4 and 8.2 kyrs cal BP to the combined effects of rapid sea level rise and subsequent flooding following Lake Agassiz’s freshwater outburst and the cool and dry climatic conditions of the 8.2 kyrs cal BP event caused by a reduced North Atlantic thermohaline circulation. Our well-constrained chronology of rapid salinity changes in the Northern Aegean Sea might assist in unravelling the longstanding discussion of catastrophic sea-level changes impeding the Neolithisation of southeastern Europe.

## Methods

### Sediment core SL152

This study focused on the Holocene sediment gravity core GeoTÜ SL152 (40°05.19′N, 24° 36.65′E; water depth: 978 m) recovered in 2001 during R.V. Meteor cruise M51/3 from the Mount Athos Basin, northern Aegean Sea. The hemipelagic muds are rapidly deposited (about 31–37 cm per thousand years) including an organic-rich layer, the so-called sapropel 1 (S1). The position of the core SL152 approximately 130 km west from Sedd el Bahr at the opening of the Marmara Sea is ideal to record the outflow of low salinity Black Sea surface water into the Northern Aegean Sea through the Dardanelles-Bosporus corridor.

### Age model

**Figure 3:**
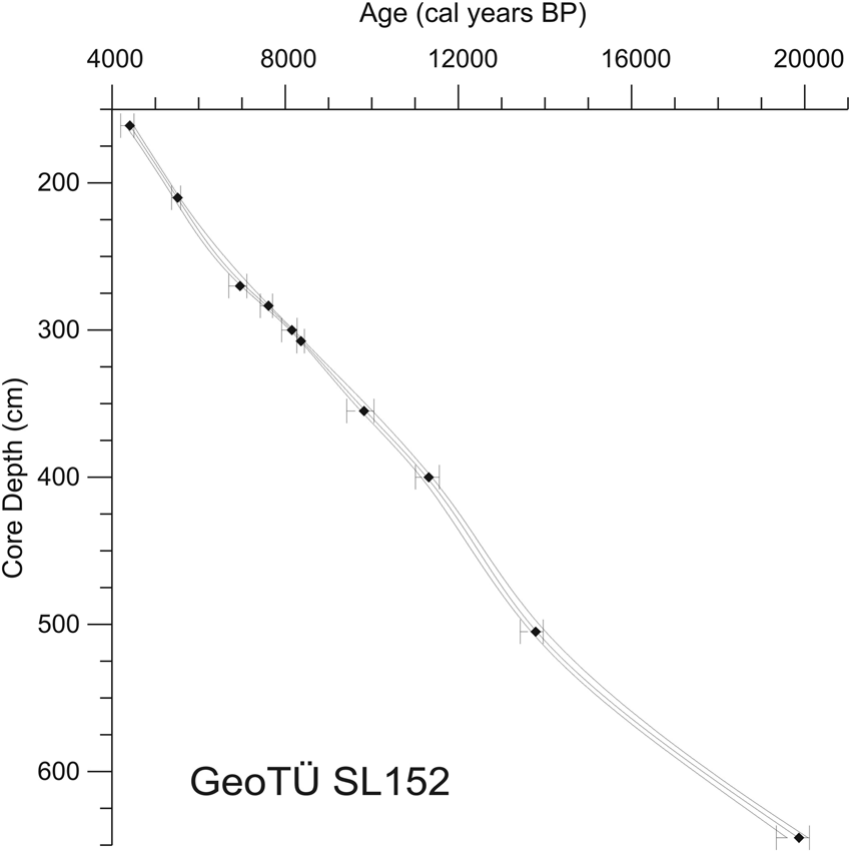
Revised age model for core GeoTü SL152 of reference^9^. Age-depth graph for the six ^14^C AMS radiocarbon ages (210–355.5 cm, see Supplement Table 1) embedded vs. core depth in core GeoTÜ SL152.

The chronology of core GeoTÜ SL152 along with the sapropel 1 (S1) section is based on six accelerator mass spectrometry ^14^C dates. Four AMS dates were taken from ref.^9^ in 10/2005 and two new dates (sampled in 05/2016) were taken for the present study (Supplement Data Table 1,2, Fig. 3). The ages of ref.^9^ are from tests of the planktic foraminifera *Globigerinoides ruber* and *G. bulloides* (Supplement Data Table 1). Our new ages are based on a mixed planktic foraminifera all in the limited size fraction of >200 µm. The ^14^C analyses were performed at the Leibniz Laboratory for Radiometric Dating and Stable Isotope Research, Kiel and at Beta Analytic Inc. in Florida (new samples Beta-483196 and Beta-483197 (Supplement Data Table 1). All conventional radiocarbon ages had sigma errors between 30 and 55 years, and were converted to calendar years with a local reservoir correction ΔR of-113 years and the MARINE13 database^33^. For age modeling and correction, we used the software program Clam^34^ with a spline-fit model based on 10000 iterations and the default smoothing level of 0.3. AMS calendar years are expressed as best and min/max ages (95% probability). Our age-depth curve shows that the six ^14^C dates yield highly consistent ages (Fig. 3). Changing sedimentation rates across our studied interval are only minor (Supplement Data Table 2).

### Phytoplankton-based reconstruction of Sea surface Salinity (SSS)

To calculate mean annual SSS we used the methods as outline by ref.^13^. All SSS estimates presented here are based on transfer-function subset 4 of ref.^13^ in which all coccoliths of *Emiliania huxleyi* larger than 4 µm were excluded. Our results are presented as deviation from the average mean annual SSS of the presented interval (sea surface salinity anomaly, Supplement Data Table 2), in practical salinity units to record relative SSS anomalies. The 1 psu SSS anomaly recorded over our record at site SL152 corresponds to a 1 per mil change in δ^18^O_seawater_ at site LC21 across the same period. As δ^18^O_seawater_ is effectively a record of the (isotopically lighter) freshwater budget^14^, this provides independent corroboration of our phytoplankton-derived salinity proxy.

We analyzed in total 47 samples for reconstructing SSS changes with a centennial to decadal time resolution. About 50 flat lying placoliths of *E. huxleyi* per sample were digitized using a ZEISS SIGMA scanning electron microscope at a magnification of 20,000X and measured using the software ImageJ. The image size was 1024 * 768 pixels. The dimensions of the images were calibrated by measuring 30 mono-sized polymer calibration spheres with a diameter of 1.998 ± 0.016 µm (Duke Standard) for each sample. All measurements for calculation SSS are shown in Supplement Data Table 2.

### Data availability

Data related to this paper may be requested from the corresponding author.

## Electronic supplementary material

Supplementary information
