# Supplementary material for: Black Sea outflow response to Holocene meltwater events
Source: Sci Rep. 2018 Mar 6;8:4081. doi: 10.1038/s41598-018-22453-z (PMC5840179; doi:10.1038/s41598-018-22453-z)
Supplement: Supplementary file 1 — Supplementary information [file 41598_2018_22453_MOESM1_ESM.pdf]

Black Sea outflow response to Holocene meltwater events

Jens O. Herrle[1,2]*, Jörg Bollmann[3], Christina Gebühr[1,2], Hartmut Schulz[4], Rosie M. Sheward[1], Annika Giesenberg[1]

Supplement Table 1. Table of dated materials, AMS-^{14}C ages, corrections and conversions into calendar ages (bold ^{14}C ages measured in this study all other ^{14}C ages are taken from reference 9, PF = planktic foraminifera, *G. ruber* = *Globigerinoides ruber; G. bulloides* = *Globigerina bulloides*).

Lab_Code #	Sampled Depth Interval (cm)	Mean Sample Depth (cm)	Sampled Material Sieved size fraction	Conventional Radiocarbon Age (yr BP)	Calendar Age 2-sigma error 95% probability (Cal yr BP) this study
KIA28411	208-212	210.0	*G. ruber* >200 µm	5265±35	5516 (5444-5583)
KIA28412	268.5-272	270.3	*G. ruber* >200 µm	6570±45	6953 (6824-7109)
**Beta-438196**	**283-284**	**283.5**	**mixed PF >200 µm**	**7290±30**	**7612 (7571-7706)**
KIA28413	298-302	300.0	*G. ruber, G. bulloides* >200 µm	7805±40	8152 (8034-8273)
**Beta-438197**	**307-308**	**307.5**	**mixed PF >200 µm**	**8030±30**	**8392 (8314-8442)**
KIA28414	353-358	355.5	*G. ruber* >200 µm	9205±55	9819 (9619-10049)

Supplement Table 2. Measurements of *E. huxleyi* placoliths, sedimentation rate, and calculated sea surface salinity and anomaly of core SL152 (psu = practical salinity units).

Sample No.	Core depth (top cm)	Core depth (bottom cm)	Best age (cal BP)	Sedimentation rate (cm/1000 years)	No. *E. huxleyi* coccoliths measured per sample	Mean coccolith length (μm)	Mean central area width (CAW, μm)	Mean central area area, (CAA, μm^2)	Sea surface salinity (psu), plankton calibration subset 4	Sea surface salinity anomaly (psu)
SL152 261	190.0	191.0	5096	22.32	42	3.62	0.84	1.28	38.35	0.70
SL152 262	195.0	196.0	5185	22.23	41	3.60	0.84	1.26	38.07	0.42
SL152 263	200.0	201.0	5296	22.12	47	3.68	0.83	1.27	38.67	1.02
SL152 264	205.0	206.0	5406	21.99	47	3.53	0.82	1.22	37.94	0.29
SL152 265	210.0	211.0	5516	21.84	49	3.58	0.85	1.26	37.79	0.14
SL152 266	215.0	216.0	5625	21.83	46	3.66	0.87	1.33	38.31	0.66
SL152 268	225.0	226.0	5846	22.46	44	3.68	0.86	1.32	38.50	0.85
SL152 269	230.0	231.0	5959	23.1	47	3.55	0.81	1.19	37.81	0.16
SL152 270	235.0	236.0	6077	23.95	30	3.62	0.80	1.18	38.21	0.56
SL152 271	240.0	241.0	6199	25.03	48	3.53	0.80	1.17	37.81	0.16
SL152 272	245.0	246.0	6327	26.32	32	3.67	0.84	1.26	38.29	0.64
SL152 273	250.0	251.0	6462	27.82	44	3.56	0.83	1.23	37.85	0.20
SL152 274	255.0	256.0	6605	29.54	37	3.54	0.86	1.29	37.73	0.08
SL152 275	260.0	261.0	6757	31.48	44	3.44	0.83	1.20	37.26	-0.39
SL152 276	265.0	266.0	6919	33.64	35	3.51	0.84	1.25	37.67	0.02
SL152 277	270.0	271.0	7092	35.99	45	3.52	0.87	1.32	37.76	0.11
SL152 286	275.0	275.5	7267	37.42	48	3.52	0.82	1.21	37.82	0.17
SL152 296	280.0	280.5	7455	37.46	44	3.50	0.85	1.27	37.62	-0.03
SL152 302	283.0	283.5	7566	36.76	50	3.34	0.83	1.19	36.66	-0.99
SL152 303	283.5	284.0	7585	36.61	51	3.41	0.85	1.25	37.12	-0.53
SL152 304	284.5	285.0	7621	36.29	46	3.57	0.87	1.31	37.84	0.19
SL152 305	285.0	285.5	7639	36.14	49	3.58	0.90	1.39	38.00	0.35
SL152 312	288.5	289.0	7764	35.12	46	3.41	0.86	1.27	37.07	-0.58
SL152 314	289.5	290.0	7799	34.85	51	3.43	0.83	1.21	37.31	-0.34
SL152 321	293.0	293.5	7920	33.95	50	3.51	0.85	1.27	37.65	0.00
SL152 323	294.0	294.5	7954	33.72	46	3.50	0.82	1.20	37.57	-0.08

Sample No.	Core depth (top cm)	Core depth (bottom cm)	Best age (cal BP)	Sedimentation rate (cm/1000 years)	No. *E. huxleyi* coccoliths measured per sample	Mean coccolith length (μm)	Mean central area width (CAW, μm)	Mean central area area, (CAA, μm^2)	Sea surface salinity (psu), plankton calibration subset 4	Sea surface salinity anomaly (psu)
SL152 325	295.0	295.5	7988	33.49	53	3.46	0.82	1.19	37.31	-0.34
SL152 327	296.0	296.5	8021	33.27	46	3.46	0.83	1.22	37.42	-0.23
SL152 329	297.0	297.5	8054	33.06	48	3.45	0.85	1.28	37.58	-0.07
SL152 335	300.0	300.5	8152	32.48	45	3.45	0.87	1.30	37.32	-0.33
SL152 336	300.5	301.0	8169	32.39	52	3.46	0.84	1.23	37.30	-0.35
SL152 339	302.0	302.5	8217	32.14	46	3.48	0.86	1.31	37.76	0.11
SL152 345	305.0	305.5	8313	31.75	43	3.42	0.85	1.24	37.04	-0.61
SL152 349	307.0	307.5	8376	31.55	52	3.29	0.83	1.18	36.45	-1.20
SL152 356	310.5	311.0	8486	31.27	56	3.34	0.84	1.21	36.70	-0.95
SL152 361	313.0	313.5	8565	31.11	51	3.36	0.86	1.28	37.11	-0.54
SL152 365	315.0	315.5	8627	30.99	51	3.38	0.88	1.33	37.21	-0.44
SL152 376	320.5	321.0	8796	30.74	43	3.51	0.85	1.29	37.82	0.17
SL152 386	325.5	326.0	8950	30.63	45	3.49	0.85	1.26	37.48	-0.17
SL152 396	330.5	331.0	9103	30.6	35	3.47	0.81	1.16	37.27	-0.38
SL152 406	335.5	336.0	9256	30.68	34	3.52	0.87	1.31	37.68	0.03
SL152 416	340.5	341.0	9410	30.84	35	3.49	0.85	1.26	37.48	-0.17
SL152 426	345.5	346.0	9564	31.11	47	3.52	0.85	1.27	37.69	0.04
SL152 433	349.0	349.5	9674	31.35	43	3.50	0.88	1.33	37.57	-0.08
SL152 436	355.0	356.0	9871	31.9	46	3.54	0.86	1.31	37.95	0.30
SL152 437	360.0	361.0	10031	32.26	51	3.58	0.90	1.39	38.00	0.35
SL152 439	370.0	371.0	10355	32.38	42	3.64	0.91	1.44	38.55	0.90
SL152 441	380.0	381.0	10676	31.72	42	3.58	0.30	0.89	38.06	0.41
